# A case of paraneoplastic optic neuropathy and outer retinitis positive for autoantibodies against collapsin response mediator protein-5, recoverin, and α-enolase

**DOI:** 10.1186/1471-2415-14-5

**Published:** 2014-01-16

**Authors:** Michiyuki Saito, Wataru Saito, Atsuhiro Kanda, Hiroshi Ohguro, Susumu Ishida

**Affiliations:** 1 Department of Ophthalmology, Hokkaido University Graduate School of Medicine, Sapporo, Japan; 2 Department of Ophthalmology, Sapporo Medical University School of Medicine, Sapporo, Japan

**Keywords:** Neuroretinitis, Recoverin, Cancer-associated retinopathy, Collapsin response mediator protein-5, Outer retinitis

## Abstract

**Background:**

Specific cross-reacting autoimmunity against recoverin or collapsin response mediator protein (CRMP)-5 is known to cause cancer-associated retinopathy or paraneoplastic optic neuropathy, respectively. We report a rare case with small cell lung carcinoma developing bilateral neuroretinitis and unilateral focal outer retinitis positive for these antibodies.

**Case presentation:**

A 67-year-old man developed bilateral neuroretinitis and foveal exudation in the right eye. Optical coherence tomography showed a dome-shaped hyperreflective lesion extending from inner nuclear layer to the photoreceptor layer at the fovea in the right eye. Single-flash electroretinography showed normal a-waves in both eyes and slightly reduced b-wave in the left eye. Results of serological screening tests for infection were within normal limits. The patient’s optic disc swelling and macular exudation rapidly improved after oral administration of prednisolone. Systemic screening detected lung small cell carcinoma and systemic chemotherapy was initiated. Immunoblot analyses using the patient’s serum detected autoantibodies against recoverin, CRMP-5, and α-enolase, but not carbonic anhydrase II. Neuroretinitis once resolved after almost remission of carcinoma on imaging but it recurred following the recurrence of carcinoma.

**Conclusions:**

The development of neuroretinitis in this cancer patient with anti-retinal and anti-optic nerve antibodies depended largely on the cancer activity, suggesting the possible involvement of paraneoplastic mechanisms. Patients with paraneoplastic optic neuropathy and retinopathy are likely to develop autoimmune responses against several antigens, thus leading to various ophthalmic involvements.

## Background

Paraneoplastic retinopathy including cancer-associated retinopathy (CAR) and paraneoplastic optic neuropathy (PON) are autoimmune diseases in which the host response to tumor antigens triggers cross-reactions to an overlapping epitope in the retina and/or the optic nerve [1]. A 23-kDa recoverin protein, localized in photoreceptors, is one of the major antigens linked with CAR [2,3]. Patients with CAR basically exhibit no abnormal retinal appearances in the initial stage and may later develop diffuse pigment epithelial degeneration similar to retinitis pigmentosa together with photoreceptor degeneration [1]. Multiple ancillary tests including immunoblot analyses using patients’ sera, systemic screening, electroretinography, and perimetry are needed for a diagnosis of CAR. As for autoantigens causing PON, collapsin response mediator protein (CRMP)-5 has been most frequently reported [4-7]. Patients with anti-CRMP-5 antibody-positive PON generally develop funduscopic features of neuroretinitis [5]. On the other hand, some patients with CAR involve inflammatory findings such as retinal vasculitis [1,8]. In this report, we describe a rare case with small cell lung carcinoma positive for anti-recoverin antibody and anti-CRMP-5 antibody presenting bilateral neuroretinitis and focal outer retinitis.

## Case presentation

**Figure 1:**
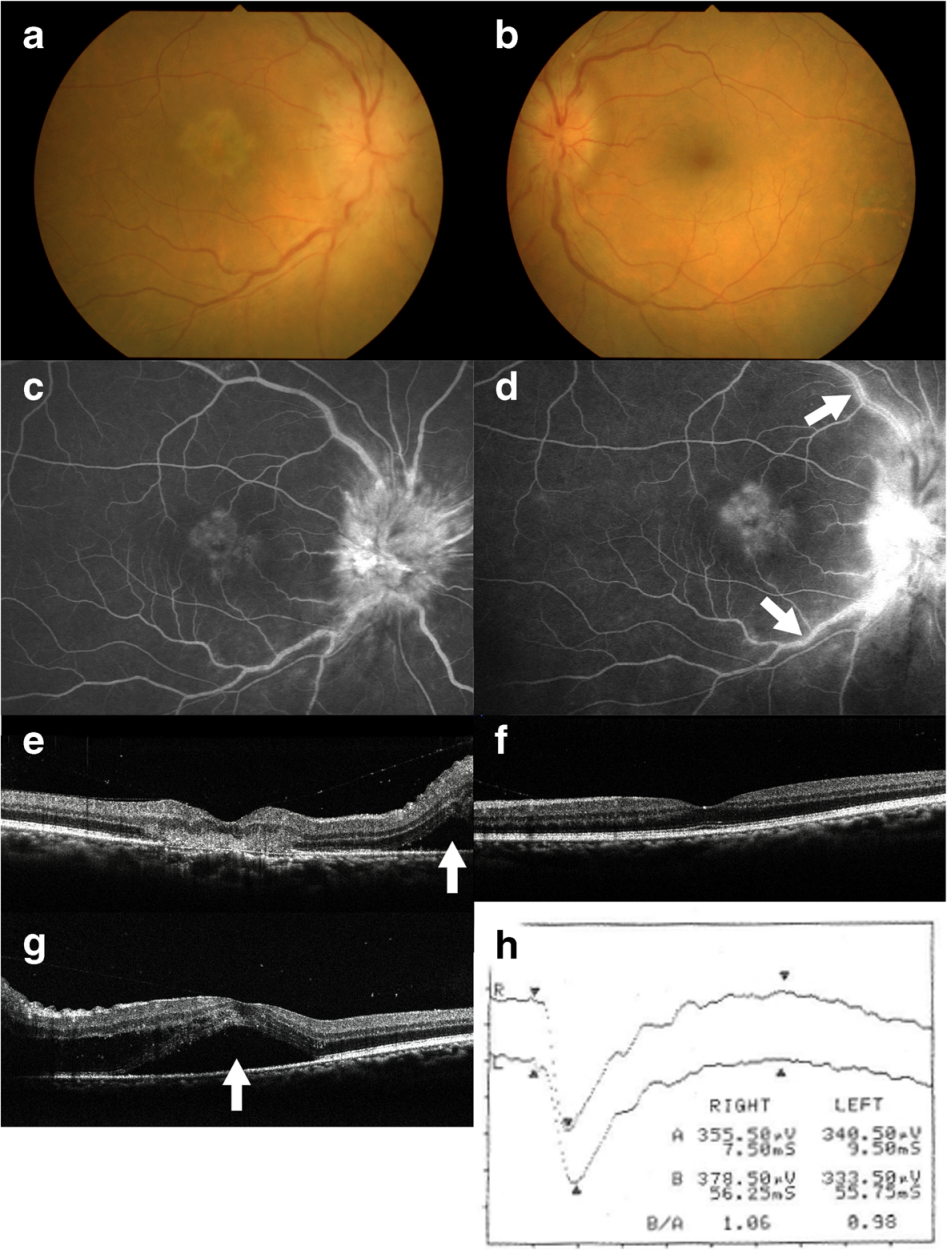
**Photographs at the first visit (a-f) and two weeks after (g, h) in a 67-year-old neuroretinitis patient with small cell lung carcinoma.** Fundus photograph showing optic disc swelling surrounded by serous retinal detachment (SRD) and dilated tortuous veins in both eyes, and a subretinal opaque exudation at the fovea in the right eye (**a**, right eye, **b**, left eye). Fluorescein angiography of the right eye showing hyperfluorecence corresponding to the foveal lesion and the optic disc 140 second after the dye injection **(c)**, and the foveal tissue staining, marked leakage from the optic disc, and retinal vasculitis (arrows) on the late phase **(d)**. Horizontal section of optical coherence tomography (OCT) showing a dome-shaped hyperreflective lesion at the fovea and SRD (**e**, arrow) adjacent to the optic disc in the right eye **(e)**. Horizontal OCT showing intact the photoreceptor inner segment/outer segment junction (IS/OS) line at the macula in the left eye **(f)**. SRD extended to the macula in the left eye (**g**, arrow). Single-flash electroretinography demonstrated normal a-wave in both eyes and slightly reduced b-wave in the left eye **(h)**.

A 67-year-old man suffered from progressive central vision loss OD for one month with no photopsia, night blindness, or extraocular symptoms including headache. The patient had medical history of pulmonary emphysema and non-contributory family history. Best-corrected visual acuity (BCVA) was 0.08 OD and 1.2 OS. Relative afferent pupillary defect (RAPD) was negative. Slit-lamp biomicroscopy showed moderate cells in the anterior vitreous OU. Funduscopy revealed swollen optic disc surrounded by serous retinal detachment (SRD), dilated tortuous veins OU, and subretinal opaque exudation at the fovea OD (Figure 1a,b). Fluorescein angiography showed initial hyperfluorescence with late leakages from the optic disc and retinal venous wall staining OU, and hyperfluorescence from the initial phase at the fovea OD (Figure 1c,d). Indocyanine green angiography revealed normal appearances OU except for slight hypofluorescence on the late phase corresponding to the foveal lesion OD. Optical coherence tomography (OCT) showed SRD adjacent to the optic disc OU and a dome-shaped hyperreflective lesion that extended from the inner nuclear layer to the photoreceptor layer corresponding to the foveal exudation OD (Figure 1e). In the left eye, the photoreceptor inner segment/outer segment junction (IS/OS) line was intact at the macula (Figure 1f). Goldmann perimetry showed blind spot enlargement of 20 × 20 OU and central scotoma of 10 × 10 OD. P100 latency of visual evoked potential (VEP) responses showed no prolongation OU (R: 111.3 ms L: 112.3 ms). Brain and orbital magnetic resonance imaging (MRI) revealed no abnormalities.

Two weeks after the first visit, BCVA decreased to 0.02 OD and 0.3 OS, with aggravation of the optic disc swelling OU and development of SRD at the macula OS (Figure 1g). Single bright-flash electroretinography (ERG) showed normal a-wave OU and slightly reduced b-wave OS (Figure 1h). Results of serological screening tests for infection, including syphilis and anti-*Bartonella henselae* antibody, as well as autoantibodies for autoimmune diseases were within normal limits.

**Figure 2:**
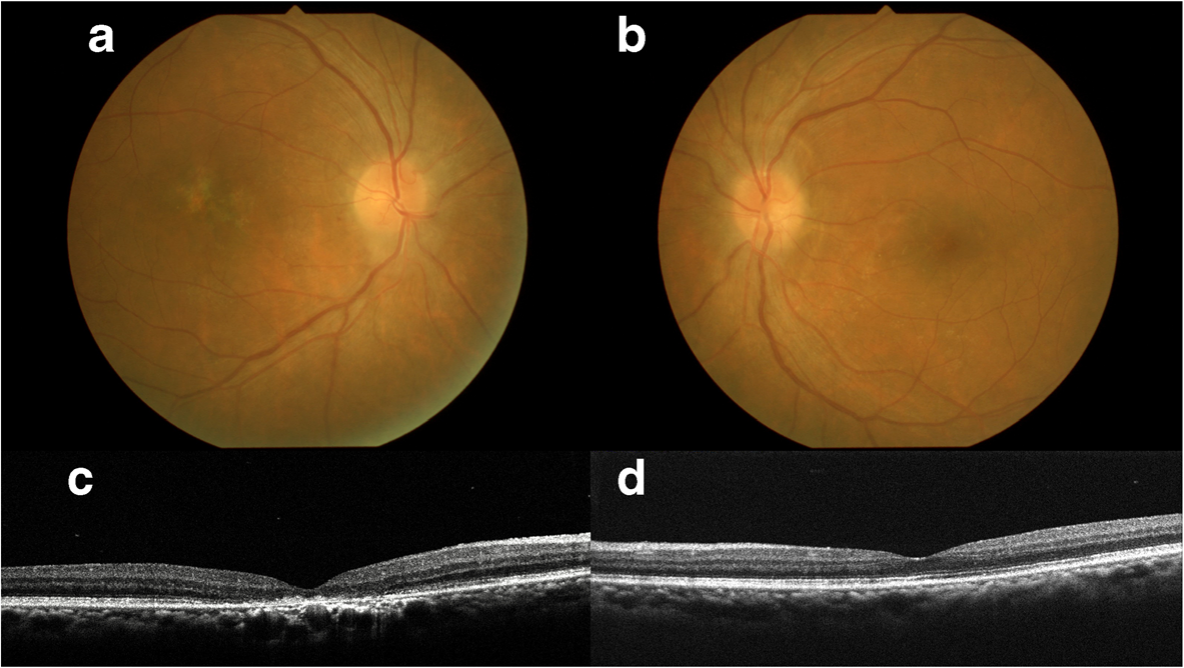
**Photographs 5 months after systemic corticosteroid treatment.** Fundus photographs showing the disappearance of the optic disc swelling and SRD in both eyes and foveal scar formation in the right eye (**a**, right eye, **b**, left eye). Horizontal OCT showing the disappearance of SRD in both eyes and a foveal hyperreflective lesion in the right eye, with intact IS/OS line in the left eye (**c**, right eye, **d**, left eye).

**Figure 3:**
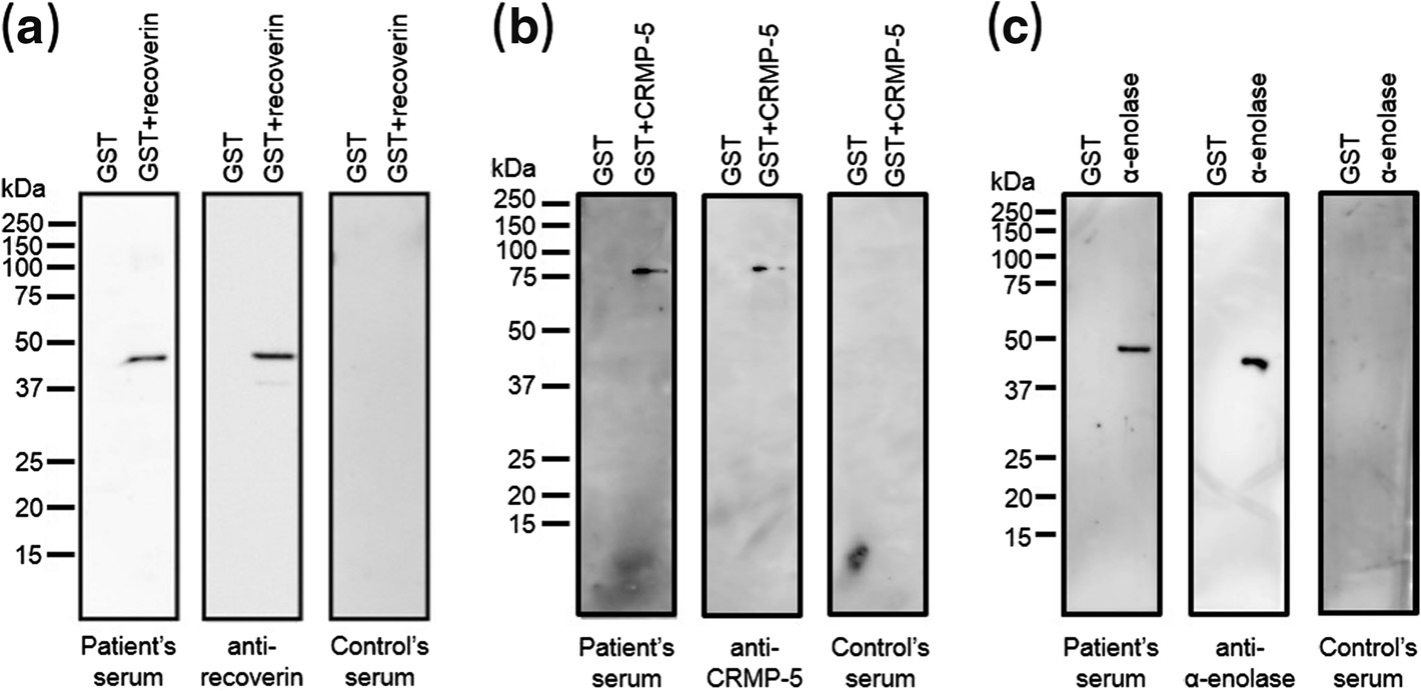
**Immunoblotting results in our patient.** Immunoblot analyses revealed predicted protein bands of approximately 49 kDa [recombinant human recoverin (23 kDa)-fusion GST (glutathione S-transferase, 26 kDa) protein] **(a)**, 88 kDa [recombinant human CRMP-5 (62 kDa)-fusion GST protein] **(b)**, and 46 kDa [recombinant human α-enolase (46 kDa)] **(c)** in the patient’s and control’s sera.

Oral administration of prednisolone (PSL) at the dose of 30 mg a day was initiated and was continued during 5 months, based on a diagnosis of bilateral neuroretinitis. Swollen optic disc and SRD quickly reduced after treatment. Systemic screening detected lung small cell carcinoma of extensive-stage disease and systemic chemotherapy was initiated. Five months after treatment, optic disc swelling disappeared OU with foveal scar formation OD (Figure 2a,b). On OCT, SRD and a foveal hyperreflective lesion disappeared with intact IS/OS line OS (Figure 2c,d). BCVA increased to 0.08 OD and 1.2 OS. Immunoblot analyses using the patient’s serum detected autoantibodies against recoverin, CRMP-5, and α-enolase (Figure 3), but not carbonic anhydrase II (data not shown). Chemotherapy was discontinued because imaging showed near-complete disappearance of lung carcinoma. One month after withdrawal of chemotherapy, lung carcinoma recurred and systemic chemotherapy was resumed. Two months after recurrence of carcinoma, optic disc swelling also recurred and oral PSL was restarted. At the last visit, 3 months after the initiation of retreatment with PSL, optic disc swelling disappeared again OU. In OCT, the IS/OS line remained intact OU except for the fovea OD. The results of single bright-flash ERG were normal OU.

### Immunoblot analyses

#### Plasmid construction and protein expression

The human *Recoverin* cDNA (GenBank No. NM_002903) was subcloned into pGEX4T-2 vector (GE Healthcare, Piscataway, NJ), and glutathione S-transferase (GST) fusion recoverin protein was expressed in *Escherichia (E.) coli* strain Rosetta-gami 2 (DE3) (Novagen, Madison, WI). GST fusion proteins were purified through binding to Glutathione-Sepharose (GE Healthcare).

#### Immunoblot analyses for recoverin, CRMP-5, α-enolase, and carbonic anhydrase II

Recombinant human CRMP-5, α-enolase, and carbonic anhydrase II proteins were purchased from Abnova (Taipei, Taiwan), Biovision (Milpitas, CA), and ATGen (Gyeonggi-do, South Korea), respectively. Proteins were solubilized in 2 × SDS (sodium dodecyl sulfate) sample buffer by heating to 100°C for 5 minutes and separated by 10% SDS-PAGE. Then, proteins were transferred to PVDF (polyvinylidene fluoride) membrane by electroblotting, and immunoblot analyses were performed using patient’s and control’s serum (1/2000 dilution), anti-recoverin antibody (1/20000, Millipore, Billerica, MA), anti-CRMP-5 antibody (1/2000, GeneTex, Irvine, CA), anti-α-enolase antibody (1/2000, Santa Cruz Biotechnology, Santa Cruz, CA), and anti-carbonic anhydrase II antibody (1/2000, Abcam, Cambridge, MA), as previously described [9].

## Discussion

Bilateral neuroretinitis with unilateral focal outer retinitis developed in a cancer patient positive for autoantibodies against recoverin, CRMP-5, and α-enolase. The ocular manifestations depended largely on comorbid cancer activity, suggesting the possible involvement of paraneoplastic mechanisms in the ocular disorder.

Neuroretinitis is an inflammatory disorder characterized by optic disc edema and the surrounding exudation despite with or without RAPD [10]. In the differential diagnosis, papilledema and optic disc tumors were eliminated owing to the lack of abnormal brain or orbital MRI findings. Hypertensive retinopathy was also denied based on the absence of systemic hypertension. Anterior ischemic optic neuropathy was differentiated based on negative RAPD and the presence of anterior vitreous cells and retinal vasculitis. Vogt-Koyanagi-Harada disease was excluded because the patient had no extraocular symptoms of the disease and indocyanine green angiography showed no choroidal inflammation, such as multiple hypofluorescent spots that appear during the middle phase. Infectious neuroretinitis was denied because of the negative serological screening results including *Bartonella henselae*.

The pathogenesis of neuroretinitis is generally regarded as vasculitis at the optic disc [10]. In this patient, a possible cause of optic disc swelling was thought to be vasculitis rather than neuritis because of several findings such as negative RAPD, normal P100 latency in VEP, and blind spot enlargement in perimetry. Anti-CRMP-5 antibody-positive PON presents optic neuritis or neuroretinitis characterized by optic disc swelling, the leakages of the optic disc and retinal vessels on FA, and anterior vitreous cells [4,5]. Since the CRMP-5 protein localizes at oligodendrocytes within the myelin sheath of the optic nerve [6], bilateral neuroretinitis in this patient is thought to result from inflammation at the vicinity of the optic nerve caused by CRMP-5-related autoimmunity.

The present case also involved unilateral focal outer retinal inflammation with suspected fibrin formation at the fovea, which quickly responded to systemic corticosteroid therapy and completely resolved with the remaining loss of the IS/OS line. To our knowledge, no previous reports have shown such findings in patients with anti-CRMP-5 antibody-positive PON [4-7]. Reasonably, autoantibodies other than anti-CRMP-5 antibody were likely to play a role in the pathogenesis of the outer retinal inflammation.

Our case presented with not only the anti-CRMP-5 antibody but also the antibody for recoverin, a major cause of CAR. To our knowledge, only one case with both antibodies has been reported [11]. However, the ophthalmic findings in this case differed from those presented here; the former case had no initial retinal or optic disc abnormalities and later developed optic disc pallor [11]. Basically, anti-recoverin antibody-positive CAR patients have diffuse photoreceptor damage in ERG and OCT [12]. In the present case, however, photoreceptors were preserved except for the fovea OD from these OCT findings and preserved a-wave amplitude on ERG, suggesting an underlying cause distinctly different from typical cases with anti-recoverin antibody-positive CAR.

Recoverin is reported to be highly uveitogenic and antigenic in rodents and cause experimental autoimmune uveitis (EAU) together with recoverin-specific autoantibody induction and severe photoreceptor degeneration [13,14]. The histopathological analysis in a recoverin-induced EAU has demonstrated focal cell infiltration from the level of the photoreceptor to the outer plexiform layers in the early stage, which were similar to the OCT images of focal outer retinal inflammation in this case (Figure 1e). Moreover, all the aspects of recoverin-induced EAU and photoreceptor degeneration could be reproduced in naïve animals by the adoptive transfer of stimulated lymphocytes from animals previously immunized with recoverin, suggesting a possible association of cellular immunity with the recoverin-induced ocular disorder [13].

Regarding the pathogenesis by which anti-recoverin antibody causes CAR, it has been demonstrated that the antibody triggers photoreceptor cell death through apoptosis [2,15]. However, the association of T cell-mediated autoimmunity in CAR remains elusive. A case with CAR-like disease with no malignancy (benign Warthin tumor) was reported to develop bilateral vitritis, optic disc pallor and retinal vascular sheathing in addition to the typical CAR-related sign of non-recordable ERG [16]. Importantly, lymphocyte proliferative responses demonstrated a strong cellular reaction to recoverin, suggesting the validity of recoverin-specific autoimmunity in the pathogenesis of this CAR-like disease, which was then termed “recoverin-associated retinopathy (RAR)” [16]. We have reported the case of a benign tumor with anti-recoverin antibody-positive retinopathy manifesting typical CAR (i.e., photoreceptor degeneration) and retinal vasculitis with macular edema. Surprisingly, colonic adenoma excised from the patient was potently immunopositive for recoverin, leading us to advocate “benign tumor-associated retinopathy (BAR)” [17].

Thus, the recoverin-mediated autoimmune retinopathies (CAR, RAR and BAR) [8,16,17] and ophthalmic findings in the present case potentially harbor inflammatory features; however, in this cancer patient, it remains unclear why photoreceptors were mostly intact despite the induction of antirecoverin antibody. Future research is needed to elucidate the molecular and cellular mechanisms underlying recoverin-associated pathogenesis of vascular inflammation and neurodegeneration.

α-enolase is another antigen that causes CAR or PON, as this protein localizes at both the retina and the optic nerve [6]. Anti-α-enolase antibody-positive CAR patients have relatively mild clinical course that varied from stability to years to slow progression [18], suggesting its weaker pathogenicity than anti-recoverin antibody. Therefore, this antibody might contribute less than the other antibody present to the development of this patient’s ophthalmic findings.

## Conclusion

In conclusion, we encountered a case with small cell lung carcinoma that was thought to be paraneoplastic neuroretinitis and focal outer retinitis. Patients with paraneoplastic optic neuropathy and retinopathy are likely to develop autoimmune responses against several antigens, as shown in the present case, thus leading to various ophthalmic involvements.

### Consent

Written informed consent was obtained from the next of kin of the patient for publication of this case report and any accompanying images. A copy of the written consent is available for review by the Editor-in-Chief of this journal.

## Competing interests

The authors declare that they have no competing interests.

## Authors’ contributions

MS and WS contributed to conception and design of the study, the collection, analysis, and interpretation of the data, and drafting the manuscript. AK carried out the immunoblot analyses and drafted the manuscript. HO carried out the immunoblot analyses. SI contributed to conception and design of the study, and drafting the manuscript. All authors read and approved the final manuscript.

## Pre-publication history

The pre-publication history for this paper can be accessed here:

http://www.biomedcentral.com/1471-2415/14/5/prepub
